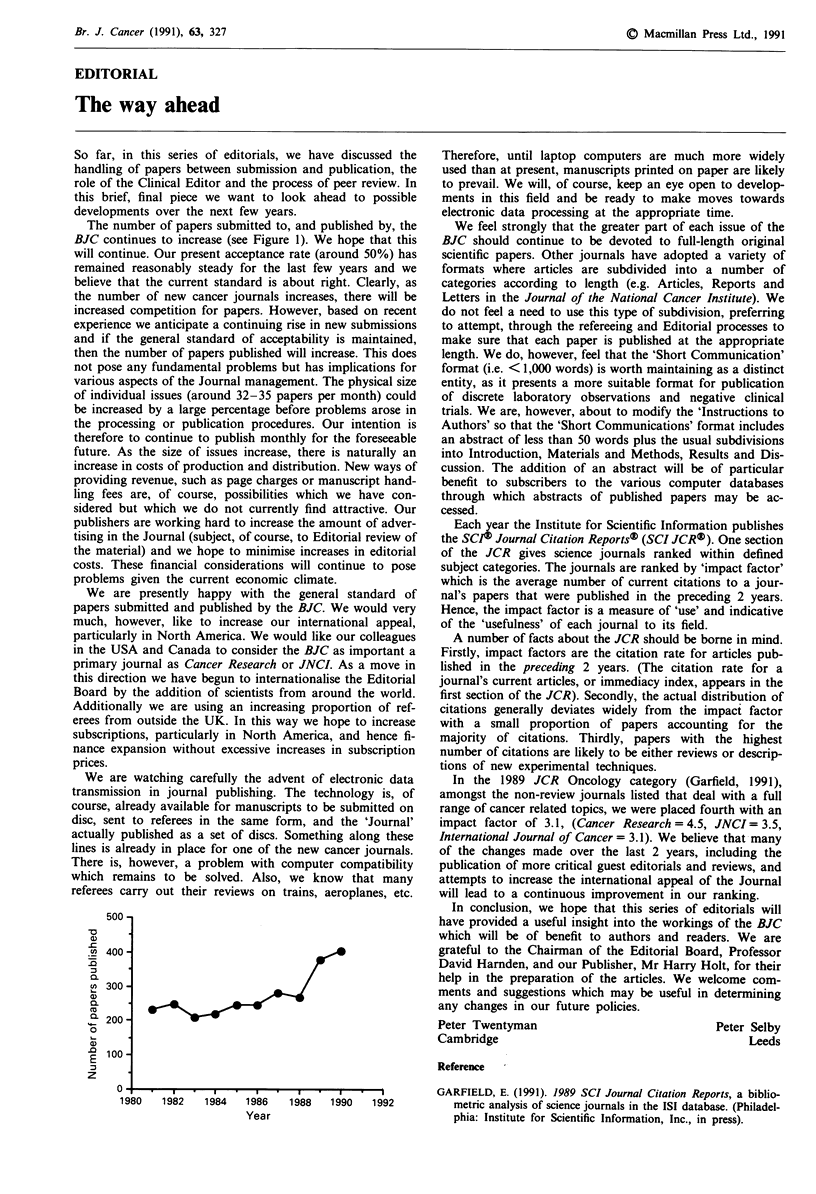# The way ahead

**Published:** 1991-03

**Authors:** Peter Twentyman, Peter Selby


© Macmillan Press Ltd., 1991

### EDITORIAL

### The way ahead

So far, in this series of editorials, we have discussed the handling of papers between submission and publication, the role of the Clinical Editor and the process of peer review. In this brief, final piece we want to look ahead to possible developments over the next few years.

The number of papers submitted to, and published by, the *BJC* continues to increase (see Figure 1). We hope that this will continue. Our present acceptance rate (around 50%) has remained reasonably steady for the last few years and we believe that the current standard is about right. Clearly, as the number of new cancer journals increases, there will be increased competition for papers. However, based on recent experience we anticipate a continuing rise in new submissions and if the general standard of acceptability is maintained, then the number of papers published will increase. This does not pose any fundamental problems but has implications for various aspects of the Journal management. The physical size of individual issues (around 32–35 papers per month) could be increased by a large percentage before problems arose in the processing or publication procedures. Our intention is therefore to continue to publish monthly for the foreseeable future. As the size of issues increase, there is naturally an increase in costs of production and distribution. New ways of providing revenue, such as page charges or manuscript handling fees are, of course, possibilities which we have considered but which we do not currently find attractive. Our publishers are working hard to increase the amount of advertising in the Journal (subject, of course, to Editorial review of the material) and we hope to minimise increases in editorial costs. These financial considerations will continue to pose problems given the current economic climate.

We are presently happy with the general standard of papers submitted and published by the *BJC*. We would very much, however, like to increase our international appeal, particularly in North America. We would like our colleagues in the USA and Canada to consider the *BJC* as important a primary journal as *Cancer Research* or *JNCI*. As a move in this direction we have begun to internationalise the Editorial Board by the addition of scientists from around the world. Additionally we are using an increasing proportion of referees from outside the UK. In this way we hope to increase subscriptions, particularly in North America, and hence finance expansion without excessive increases in subscription prices.

We are watching carefully the advent of electronic data transmission in journal publishing. The technology is, of course, already available for manuscripts to be submitted on disc, sent to referees in the same form, and the 'Journal' actually published as a set of discs. Something along these lines is already in place for one of the new cancer journals. There is, however, a problem with computer compatibility which remains to be solved. Also, we know that many referees carry out their reviews on trains, aeroplanes, etc.

Therefore, until laptop computers are much more widely used than at present, manuscripts printed on paper are likely to prevail. We will, of course, keep an eye open to developments in this field and be ready to make moves towards electronic data processing at the appropriate time.

We feel strongly that the greater part of each issue of the *BJC* should continue to be devoted to full-length original scientific papers. Other journals have adopted a variety of formats where articles are subdivided into a number of categories according to length (e.g. Articles, Reports and Letters in the *Journal of the National Cancer Institute*). We do not feel a need to use this type of subdivision, preferring to attempt, through the refereeing and Editorial processes to make sure that each paper is published at the appropriate length. We do, however, feel that the 'Short Communication' format (i.e. $<$ 1,000 words) is worth maintaining as a distinct entity, as it presents a more suitable format for publication of discrete laboratory observations and negative clinical trials. We are, however, about to modify the 'Instructions to Authors' so that the 'Short Communications' format includes an abstract of less than 50 words plus the usual subdivisions into Introduction, Materials and Methods, Results and Discussion. The addition of an abstract will be of particular benefit to subscribers to the various computer databases through which abstracts of published papers may be accessed.

Each year the Institute for Scientific Information publishes the *SCI® Journal Citation Reports®* (*SCI JCR®*). One section of the *JCR* gives science journals ranked within defined subject categories. The journals are ranked by 'impact factor' which is the average number of current citations to a journal's papers that were published in the preceding 2 years. Hence, the impact factor is a measure of 'use' and indicative of the 'usefulness' of each journal to its field.

A number of facts about the *JCR* should be borne in mind. Firstly, impact factors are the citation rate for articles published in the *preceding* 2 years. (The citation rate for a journal's current articles, or immediacy index, appears in the first section of the *JCR*). Secondly, the actual distribution of citations generally deviates widely from the impact factor with a small proportion of papers accounting for the majority of citations. Thirdly, papers with the highest number of citations are likely to be either reviews or descriptions of new experimental techniques.

In the 1989 *JCR* Oncology category (Garfield, 1991), amongst the non-review journals listed that deal with a full range of cancer related topics, we were placed fourth with an impact factor of 3.1, (*Cancer Research* = 4.5, *JNCI* = 3.5, *International Journal of Cancer* = 3.1). We believe that many of the changes made over the last 2 years, including the publication of more critical guest editorials and reviews, and attempts to increase the international appeal of the Journal will lead to a continuous improvement in our ranking.

In conclusion, we hope that this series of editorials will have provided a useful insight into the workings of the *BJC* which will be of benefit to authors and readers. We are grateful to the Chairman of the Editorial Board, Professor David Harnden, and our Publisher, Mr Harry Holt, for their help in the preparation of the articles. We welcome comments and suggestions which may be useful in determining any changes in our future policies.

Peter Twentyman

Cambridge

Peter Selby

Leeds

**Reference**

GARFIELD, E. (1991). *1989 SCI Journal Citation Reports*, a bibliometric analysis of science journals in the ISI database. (Philadelphia: Institute for Scientific Information, Inc., in press).